# DSF-BRNet: Dual-Gated Semantic Fusion and Boundary Refinement for Efficient Endoscopic Polyp Segmentation

**DOI:** 10.3390/s26092717

**Published:** 2026-04-28

**Authors:** Botao Liu, Changqi Shi, Ming Zhao

**Affiliations:** 1 School of Computer Science, Yangtze University, Jingzhou 434023, China; 500327@yangtzeu.edu.cn (B.L.); shichangqi17@gmail.com (C.S.); 2 School of Cyber Science and Engineering, Wuxi University, Wuxi 214105, China

**Keywords:** polyp segmentation, efficient network, real-time segmentation, semantic fusion, boundary refinement

## Abstract

Early detection and accurate segmentation of colorectal polyps during colonoscopy are crucial for the prevention of colorectal cancer. However, automated polyp segmentation remains challenging because of high inter-class variance, complex intestinal backgrounds, and blurred boundaries. To address these issues while maintaining computational efficiency, DSF-BRNet was developed for endoscopic polyp segmentation. In this framework, a Dual-Gated Semantic Fusion (DSF) module is introduced to reduce spatial misalignment between cross-level features and to provide a more reliable semantic basis for lesion localization. To further alleviate boundary ambiguity, a High-Frequency Boundary Refinement (HBR) module is used to sharpen segmentation contours under aligned semantic guidance. Together, these components form an Align-then-Refine framework in which semantic localization is strengthened before boundary refinement is performed. Experiments on four public benchmark datasets—Kvasir-SEG, CVC-ClinicDB, CVC-ColonDB, and ETIS-LaribPolypDB—showed competitive performance with favorable computational efficiency. Mean Dice scores of 0.943 on CVC-ClinicDB and 0.818 on ETIS-LaribPolypDB were achieved, with 25.55 M parameters and an inference speed of 80.08 FPS. These results indicate that accurate semantic localization and fine boundary preservation can be achieved simultaneously, suggesting that the method may be promising for real-time computer-aided diagnosis (CAD).

## 1. Introduction

Colorectal cancer (CRC) is one of the most common and lethal malignancies globally, posing a severe threat to public health [1,2]. Clinical studies have extensively demonstrated that the early detection and resection of precancerous colorectal polyps during colonoscopy can significantly reduce the mortality rate associated with CRC [3,4]. However, traditional manual screening highly depends on the experience and concentration of the endoscopists. Due to the prolonged operation time and visual fatigue, the polyp miss rate remains non-negligible, particularly for small or flat polyps that are camouflaged within the intestinal mucosa [5,6]. Consequently, developing an automated, robust, and accurate computer-aided diagnosis system for endoscopic polyp segmentation is of paramount clinical significance.

In recent years, deep learning models have significantly advanced automatic polyp segmentation [7]. Early architectures like U-Net [8] and UNet++ [9] rely on encoder–decoder structures with skip connections, but they struggle with the extreme scale variations and diverse morphologies of polyps. To address this, task-specific models have been proposed; for instance, PraNet [10] utilizes reverse attention for boundary mining, while Polyp-PVT [11] employs a transformer backbone to capture long-range dependencies.

Despite the promising performance of these task-specific networks, automated polyp segmentation remains highly challenging due to two primary obstacles: (1) Semantic Gap and Spatial Misalignment: High-level features contain rich semantics but lack spatial resolution, whereas low-level features possess fine details but are easily contaminated by background noise [12]. Directly fusing these cross-level features often causes spatial misalignment and false positives in complex mucosal backgrounds. (2) Boundary Ambiguity: Due to high intra-class variance and low contrast between polyps and surrounding tissues, precise contour delineation remains a bottleneck [13,14]. To explicitly tackle these intertwined challenges, various boundary-enhancement techniques have been explored [15]. However, rigorous experimental investigation reveals a critical underlying mechanism: boundary refinement is largely ineffective, or even counterproductive, when fundamental semantic features are spatially misaligned. Aggressively injecting high-frequency edge cues without precise semantic localization tends to amplify background noise and generate misplaced boundaries. Therefore, the main issue addressed in this paper is the mismatch between reliable semantic localization and accurate boundary recovery in challenging endoscopic scenes.

To overcome these accuracy bottlenecks, one might look toward the recent emergence of vision foundation models, which have demonstrated remarkable zero-shot generalization capabilities. However, a critical clinical dilemma persists: their staggering parameter counts (often exceeding hundreds of millions) and massive memory consumption render high-framerate real-time inference practically unattainable on the resource-constrained edge computing platforms of current clinical endoscopy towers. Consequently, achieving a pragmatic balance between lightweight architecture and robust accuracy remains a pressing necessity for real-world clinical deployment.

To address this issue, DSF-BRNet, a Dual-Gated Semantic Fusion and Boundary Refinement Network, was developed for efficient endoscopic polyp segmentation. The framework follows a sequential Align-then-Refine strategy, in which semantic misalignment is reduced before contour sharpening is performed. A Dual-Gated Semantic Fusion (DSF) module is used to model channel-wise and spatial relationships for cross-level alignment, thereby providing a more reliable semantic basis. On this basis, a High-Frequency Boundary Refinement (HBR) module employs a fixed-weight Sobel operator to extract structural cues, which are fused with aligned semantics through learnable weights to refine polyp contours without introducing a notable computational burden. In summary, the main contributions of this paper are four-fold:

1. A novel and efficient deep learning network, termed DSF-BRNet, is presented for polyp segmentation, achieving a favorable balance between efficiency and accuracy while addressing the challenges of complex intestinal backgrounds and blurred boundaries.
2. The Dual-Gated Semantic Fusion (DSF) module is designed to effectively correct spatial misalignments between hierarchical features, establishing a precise semantic localization foundation.
3. A High-Frequency Boundary Refinement (HBR) module is introduced. Guided by aligned semantics, it utilizes high-frequency structural cues to actively refine and sharpen segmentation contours.
4. An Align-then-Refine strategy is comprehensively conceptualized and validated. Extensive experiments on four public benchmark datasets demonstrate that the proposed DSF-BRNet achieves competitive accuracy with superior real-time efficiency.

The remainder of this paper is organized as follows: Section 2 reviews related work. Section 3 details the DSF-BRNet method. Section 4 presents the experiments and discussion. Section 5 concludes the paper.

## 2. Related Work

### 2.1. Deep Learning for Medical Image Segmentation

Deep learning architectures have fundamentally driven the progress of medical image analysis [16]. Early frameworks, such as Fully Convolutional Networks (FCNs) [17], formulated segmentation as a pixel-level classification task. Subsequently, U-Net established the standard encoder–decoder paradigm, utilizing skip connections to transfer low-level spatial details directly to high-level semantic maps. To further mitigate the semantic gap between encoder and decoder stages, UNet++ introduced nested and dense skip pathways. Recently, Vision Transformers [18] have been adapted for medical image segmentation to capture long-range dependencies and global contextual representations. While these general-purpose models demonstrate robust performance across various medical imaging modalities, applying them directly to colonoscopy images often yields suboptimal results due to the severe scale variations and low contrast typical of endoscopic environments.

### 2.2. Automated Polyp Segmentation

To address the unique characteristics of colorectal polyps, task-specific segmentation networks have been extensively investigated. PraNet introduced a reverse attention mechanism to systematically mine boundary cues and refine the localization of polyps. SANet [19] proposed a shallow attention module combined with a color exchange strategy to filter out background noise from low-level features. Furthermore, transformer-based architectures, such as Polyp-PVT, and adaptive multi-scale perception frameworks [20] have been utilized to extract robust global contexts, effectively handling multi-scale variations. However, when segmenting camouflaged polyps that share similar textures with the surrounding intestinal mucosa, these methods frequently suffer from false positives and ambiguous contours. This limitation primarily stems from the lack of explicit spatial alignment constraints during the aggregation of cross-level features.

### 2.3. Feature Fusion and Boundary-Aware Learning

Effective cross-layer feature fusion and precise boundary delineation are critical for accurate polyp segmentation. Conventional fusion strategies predominantly rely on direct channel concatenation or element-wise addition [21]. These operations, however, often lead to spatial misalignment and semantic dilution, given the inherent resolution and representation disparities between hierarchical levels. Furthermore, existing attention mechanisms, such as SE [22] and CBAM [23], often rely on fully connected layers, which entails severe dimensionality reduction, inevitably destroying the fine-grained spatial details crucial for medical images. To alleviate boundary ambiguity, explicit boundary-aware learning paradigms have been developed. These methods typically employ edge operators or auxiliary edge-supervision branches to explicitly constrain the network. Although beneficial for highlighting structural contours, empirical evidence indicates that blindly injecting high-frequency boundary cues without a stable semantic foundation can inadvertently amplify background noise and generate misplaced boundaries [24]. Similarly, prevalent boundary-aware methods like PraNet utilize reverse attention to implicitly erase foregrounds for edge mining, which can be fragile when dealing with severely blurred polyp contours. Related ideas have also been explored in other image segmentation tasks. For instance, a recent study on road connectivity enhancement in remote sensing imagery employed a multi-modal attention fusion strategy to improve structural integrity [25]. Although it focuses on a different application, it still suggests that feature fusion and structure-aware refinement can be beneficial in challenging segmentation scenarios. To overcome these inherent limitations, a decoupled learning paradigm is presented in this work. The proposed DSF module avoids dimensionality reduction via 1D convolutions for efficient cross-channel interaction, while the HBR module shifts from implicit erasure to explicitly injecting deterministic structural gradients, fundamentally differing from previous paradigms.

## 3. Methodology

### 3.1. Overall Architecture

**Figure 1:**
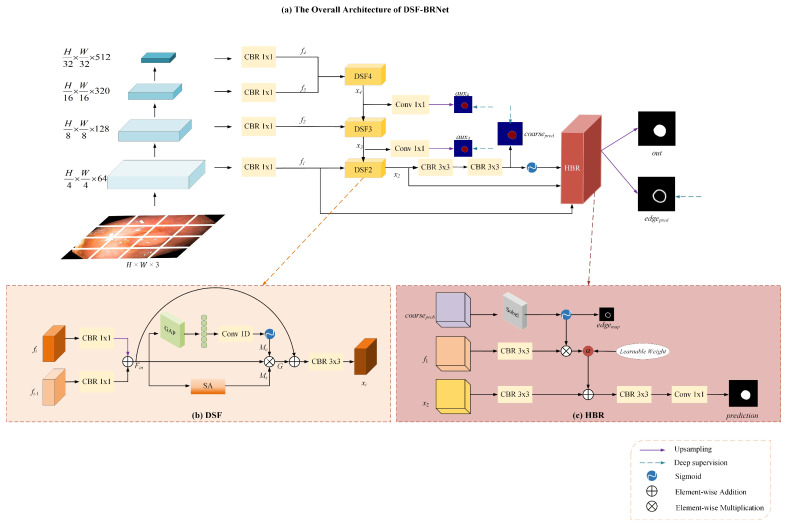
(**a**) Overall architecture of the proposed DSF-BRNet. (**b**) Architecture of DSF; (**c**) Architecture of HBR. CBR denotes Convolution+BatchNorm+ReLU.

The overall architecture of the proposed DSF-BRNet is illustrated in Figure 1a. Adopting a standard encoder–decoder paradigm, the network primarily comprises three integral components: a multi-scale feature extraction backbone, a top-down Dual-Gated Semantic Fusion (DSF) cascade, and a High-Frequency Boundary Refinement (HBR) module.

Given an input image $I\in R^{H\times W\times 3}$, the Pyramid Vision Transformer v2 (PVTv2) [26] is initially employed as the hierarchical backbone to extract multi-scale features at four distinct stages. Its pyramid structure naturally produces hierarchical representations at different resolutions, which fits well with the proposed top-down DSF cascade for cross-level feature fusion. At the same time, it provides strong contextual modeling while maintaining relatively high efficiency, making it suitable for polyp segmentation where both multi-scale representation and real-time inference are important. To streamline the computational cost and unify the channel dimensions for subsequent feature aggregation, a 1 × 1 Convolution-BatchNorm-ReLU (CBR) block is applied to each encoder stage. This operation yields a set of channel-reduced features denoted ${\left\{f_{i}\right\}}_{i=1}^{4}$, with spatial resolutions ranging from $\frac{H}{4}\times \frac{W}{4}$ to $\frac{H}{32}\times \frac{W}{32}$.

As shown in Figure 1a, the extracted backbone features are not fused by direct skip concatenation. Instead, they are progressively integrated through the top-down DSF cascade, where higher-level semantic features interact with lower-level features stage by stage. In this way, multi-scale features from different backbone stages are used consistently during decoding. The final coarse prediction, together with the shallow feature and the aligned semantic feature, is then sent to HBR for boundary refinement.

In the decoding phase, directly concatenating features from different levels often leads to semantic dilution. To address this, the DSF modules are deployed in a cascaded top-down manner to progressively aggregate the high-level semantic features and the corresponding low-level spatial features. This cascaded decoding process generates a series of semantically aligned intermediate features, denoted as $x_{4}$, $x_{3}$, and $x_{2}$. To facilitate the training process and enhance gradient propagation, deep supervision is introduced by generating auxiliary predictions $\left({aux}_{4},{aux}_{3}\right)$ from $x_{4}$ and $x_{3}$ via 1 × 1 convolutions.

Subsequently, the fused feature $x_{2}$ is passed through consecutive 3 × 3 CBR blocks and a Sigmoid function to produce a coarse segmentation probability map $coarse_{pred}$. Finally, to explicitly tackle the boundary ambiguity prevalent in endoscopic images, $coarse_{pred}$, the shallow spatial feature $f_{1}$, and the aligned semantic feature $x_{2}$ are fed into the HBR module. The HBR module dynamically extracts edge representations $edge_{pred}$ and sharpens the segmentation contours, yielding the final accurate prediction out.

### 3.2. Dual-Gated Semantic Fusion (DSF) Module

In conventional U-shaped networks, cross-layer features are typically fused via simple element-wise addition or channel concatenation. However, due to the inherent semantic gap—where high-level features possess strong semantics but low spatial resolution, while low-level features contain rich detail but abundant background noise—direct fusion frequently causes spatial misalignment. To mitigate this issue, the Dual-Gated Semantic Fusion (DSF) module is designed to recalibrate the fused features along both channel and spatial dimensions before passing them to the next stage.

As depicted in Figure 1b, let $f_{i}$ represent the high-level input feature and $f_{i-1}$ denote the low-level input feature. Initially, both features undergo a 1 × 1 CBR block to align their representations, followed by an element-wise addition to generate the initial fused feature $F_{in}$:

(1) $$F_{in}={CBR}_{1\times 1}\left(f_{i}\right)⊕{CBR}_{1\times 1}\left(f_{i-1}\right)$$

where ⊕ denotes element-wise addition. Note that ${CBR}_{1\times 1}\left(f_{i}\right)$ is first upsampled to match the spatial resolution of $f_{i-1}$ via bilinear interpolation before the addition operation.

To selectively emphasize informative regions and suppress background noise, the fused feature is subsequently routed into two parallel recalibration branches. For the channel dimension, the Efficient Channel Attention (ECA) mechanism [27] is employed instead of the fully connected design used in standard attention modules, which often introduces destructive dimensionality reduction. A Global Average Pooling (GAP) operation is first applied to aggregate the spatial information into a channel descriptor. A 1D convolution is then used to model channel-wise dependencies without compressing the channel dimension, followed by a Sigmoid activation to generate the channel attention map: $F_{in\;}{\;F}_{in\;}{\;M}_{c}$

(2) $$M_{c}=\sigma (Conv1D\left(GAP\left(F_{in}\right)\right))$$

where σ represents the Sigmoid function.

Concurrently, along the spatial dimension, a spatial attention mechanism is applied to $F_{in}$ to highlight the positional correspondences of the polyp regions. Average-pooling and max-pooling operations are first applied independently along the channel axis of $F_{in}$ to generate two 2D spatial maps. These maps are then concatenated and convolved by a standard convolution layer to produce the spatial attention map $M_{s}$:

(3) $$M_{s}=\sigma \left({Conv}_{7\times 7}\left(\left[AvgPool\left(F_{in}\right);MaxPool\left(F_{in}\right)\right]\right)\right)$$

where [⋅;⋅] denotes the concatenation operation.

Finally, the attention maps $M_{c}$ and $M_{s}$ are multiplied with the initial fused feature $F_{in}$ to perform dual-gated recalibration. To preserve the integrity of the original feature flow and prevent gradient vanishing, a residual connection is incorporated. The recalibrated feature is then smoothed by a 3 × 3 CBR block to output the final aligned feature $x_{i}$:

(4) $$x_{i}={CBR}_{3\times 3}\left(F_{in}⊕\left(F_{in}⊗M_{c}⊗M_{s}\right)\right)$$

where ⊗ denotes element-wise multiplication. Through this dual-gated mechanism, the DSF module effectively corrects spatial misalignments, providing a robust and clean semantic foundation for the subsequent boundary refinement process.

### 3.3. High-Frequency Boundary Refinement (HBR) Module

While the cascaded DSF modules successfully establish a reliable semantic foundation by correcting spatial misalignments, the inherently low contrast between polyps and normal intestinal mucosa often leads to blurred segmentation boundaries. To explicitly address this degradation, a High-Frequency Boundary Refinement (HBR) module is introduced. This module performs the final contour enhancement stage, leveraging high-frequency structural cues to sharpen contours under the strict guidance of aligned semantics. As explicitly illustrated in Figure 1c, the HBR module processes three distinct inputs: the coarse probability map $coarse_{prob}$, the shallow spatial feature $f_{1}$, and the semantically aligned deep feature $x_{2}$. Initially, a Sobel operator, implemented as fixed-weight 3 × 3 convolutional kernels to allow end-to-end training, is applied to $coarse_{prob}$ to deterministically extract spatial gradients. The Sobel operator is used here as a lightweight and deterministic structural prior for boundary refinement. Because the HBR module operates on the coarse segmentation map rather than the raw image, stable gradient cues can be obtained with minimal computational overhead and without introducing additional trainable parameters. The extracted gradients are then followed by a Sigmoid activation σ to generate a normalized edge mask, denoted as $M_{edge}$:

(5) $$M_{edge}=\sigma \left(Sobel\left({coarse}_{prob}\right)\right)$$

This mask serves a dual purpose—it acts as a spatial attention map for subsequent feature filtering, and it is explicitly supervised by the ground truth edge contours ${edge}_{map}$ during training to enforce structural fidelity. Subsequently, the shallow feature $f_{1}$ undergoes a 3 × 3 CBR block for feature adaptation. The resulting feature is multiplied element-wise ⊗ by $M_{edge}$ to suppress background noise and highlight boundary-relevant details. A learnable weight parameter α is then applied to dynamically scale these isolated high-frequency features, formulated as:

(6) $$f_{edge\_scaled}=\alpha \cdot \left({CBR}_{3\times 3}\left(f_{1}\right)⊗M_{edge}\right)$$

Concurrently, the deep semantic feature $x_{2}$ is processed through a 3 × 3 CBR block. The scaled edge feature $f_{edge\_scaled}$ is injected into this semantic representation through element-wise addition ⊕. Ultimately, the fused feature passes through a final 3 × 3 CBR block and a 1 × 1 convolution to generate the final segmentation prediction:

(7) $$prediction={Conv}_{1\times 1}\left({CBR}_{3\times 3}\left({CBR}_{3\times 3}\left(x_{2}\right)⊕f_{edge\_scaled}\right)\right)$$

Through this explicit structural guidance pathway, the HBR module effectively sharpens polyp boundaries without compromising the semantic accuracy established by the preceding cascaded fusion.

### 3.4. Loss Function

To comprehensively optimize the proposed network, a hybrid loss function is formulated to simultaneously supervise the regional semantic segmentation and explicit boundary delineation. For the semantic segmentation predictions, a structure loss $L_{struct}$, originally introduced by PraNet [10] and widely adopted in recent architectures like DCANet [28], is employed. It combines a weighted Binary Cross-Entropy (wBCE) loss and an Intersection over Union (IoU) loss [10]

(8) $$L_{struct}\left(P,G\right)=L_{wBCE}\left(P,G\right)+L_{IoU}\left(P,G\right)$$

where P represents the predicted segmentation map and G denotes the corresponding ground truth mask. The wBCE loss provides pixel-level supervision while assigning higher weights to hard pixels. The IoU loss, in turn, focuses on the global structure of the segmented target and helps alleviate the foreground/background imbalance commonly encountered in endoscopic images. The structure loss is defined as the direct sum of these two terms, without introducing an additional balancing coefficient. In this way, the semantic prediction branch is jointly constrained at both the pixel level and the region level. For the boundary branch, Dice loss is adopted because the target edge map is relatively sparse, making Dice-based supervision more suitable in this setting.

Furthermore, to guide the High-Frequency Boundary Refinement (HBR) module, an explicit edge supervision mechanism is incorporated. Given that target edge maps are inherently highly sparse, a Dice loss [29] $L_{dice}$ is utilized to supervise the edge prediction ${edge}_{pred}$:

(9) $$L_{dice}\left(E,G_{edge}\right)=1-\frac{2\sum \left(E\times G_{edge}\right)+\epsilon }{\sum E+\sum G_{edge}+\epsilon }$$

where E is the predicted edge map, $G_{edge}$ is the ground truth boundary contour, and ϵ is a smoothing factor set to 10^−5^ to ensure numerical stability.

Following standard practices in deep supervision architectures, let $P=\left\{prediction,{coarse}_{prob},{aux}_{3},{aux}_{4}\right\}$ denote the set of multi-level semantic segmentation outputs generated by the network. The total loss function $L_{total}$ is elegantly formulated as a weighted summation of the structure losses from all side outputs and the explicit boundary loss:

(10) $$L_{total}=\sum_{P_{i}\in P}L_{struct}\left(P_{i},G\right)+L_{dice}\left({edge}_{pred},G_{edge}\right)$$

This design allows the network to optimize region-level segmentation and sparse boundary supervision at the same time, which matches the roles of the semantic prediction branch and the boundary refinement branch in our framework.

## 4. Experiments

### 4.1. Evaluation Metrics

To quantitatively assess the performance of the proposed DSF-BRNet and other comparison methods, two widely adopted semantic segmentation metrics were primarily utilized: the mean Dice Similarity Coefficient (mDice) and the mean Intersection over Union (mIoU).

The Dice Similarity Coefficient calculates the spatial overlap index between the predicted segmentation mask P and the corresponding ground truth G. It places a strong penalty on false positives and false negatives, making it highly suitable for medical image segmentation tasks. mDice is formulated as:

(11) $$mDice=\frac{1}{N}\sum_{i=1}^{N}\frac{2\times \left|P_{i}\cap G_{i}\right|}{\left|P_{i}\right|+\left|G_{i}\right|}$$

where N denotes the total number of images in the testing dataset, and |⋅| calculates the number of pixels within the specified region.

Similarly, the Intersection over Union computes the ratio of the intersection area to the union area between the prediction and the ground truth. mIoU is defined as:

(12) $$mIoU=\frac{1}{N}\sum_{i=1}^{N}\frac{\left|P_{i}\cap G_{i}\right|}{\left|P_{i}\cup G_{i}\right|}$$

In addition to mDice and mIoU, which directly measure region-level similarity, other comprehensive evaluation metrics including *F-measure* $\left(F_{\beta }^{w}\right)$ [30], *S-measure* $\left(S_{\alpha }\right)$ [31] and *E-measure* $\left(E_{\phi }\right)$ [32] are also reported to evaluate boundary quality, structural similarity, and both pixel-level and image-level alignment, providing a holistic assessment of the segmentation performance.

### 4.2. Datasets

The proposed framework was evaluated on four publicly available colorectal polyp segmentation benchmark datasets to examine its robustness and generalization ability. Since these datasets differ in image quality, polyp appearance, and background complexity, they provide a suitable test bed for a more comprehensive performance assessment.

Kvasir-SEG [33]: This dataset contains 1000 high-resolution endoscopic images acquired using standard endoscopic equipment, accompanied by pixel-level ground truth masks manually annotated by expert gastroenterologists.

CVC-ClinicDB [34]: This dataset comprises 612 images extracted from 31 distinct colonoscopy video sequences. It features various polyp sizes and distinct lighting conditions, posing challenges for consistent feature extraction.

CVC-ColonDB [35]: A highly challenging dataset consisting of 380 images collected from 15 different colonoscopy sequences. It is characterized by severe variations in polyp appearance and heterogeneous intestinal backgrounds.

ETIS-LaribPolypDB [36]: This dataset contains 196 images predominantly featuring small, flat, and camouflaged polyps. It is extensively utilized as a rigorous testbed to evaluate the out-of-distribution generalization capability of models against complex mucosal structures.

Following the commonly used evaluation protocol in PraNet, 900 images from Kvasir-SEG and 550 images from CVC-ClinicDB were used for training, while the remaining 100 Kvasir-SEG images and 62 CVC-ClinicDB images were used for in-domain testing. In addition, the entire CVC-ColonDB and ETIS-LaribPolypDB datasets were used only for cross-dataset evaluation, in order to assess the generalization ability of the proposed model on unseen test sets.

### 4.3. Implementation Details

The proposed DSF-BRNet was implemented in PyTorch 2.0.0 and trained on a single NVIDIA RTX 3090 GPU. All input images were uniformly resized to 352 × 352 pixels. All images were further normalized under the same settings across all datasets. To prevent overfitting, standard data augmentations including random flipping, multi-scale rotation, and random cutout were applied. The PVTv2 backbone was initialized with ImageNet pre-trained weights, while the newly added modules were optimized via AdamW [37] with a weight decay set to 1 × 10^−5^. The network was trained for 150 epochs with a batch size of 16. An initial learning rate of 1 × 10^−4^ was employed, which decayed according to a cosine annealing schedule following a 5-epoch linear warmup. Early stopping was adopted during training. The training process was terminated when the monitored mDice did not improve for 30 consecutive epochs. All experiments were conducted with a fixed random seed.

### 4.4. Quantitative Analysis

To comprehensively evaluate the effectiveness of the proposed DSF-BRNet, it was compared against seven representative medical image segmentation models. The comparison baseline included classic encoder–decoder architectures such as U-Net [8] and UNet++ [9] alongside specialized polyp segmentation networks including PraNet [10], EU-Net [38], SANet [19], Polyp-PVT [11], and PraNetV2 [39]. For comparison, the results of several representative methods, including PraNet, EU-Net, SANet, and Polyp-PVT, were taken from the literature under the same dataset split protocol adopted in this study. The remaining methods were re-implemented and evaluated under the same experimental setting. The quantitative evaluation results across the four benchmark datasets are detailed comprehensively in Table 1, Table 2, Table 3 and Table 4. Table 1 and Table 2 show that DSF-BRNet achieved mDice values of 0.933 and 0.943 on Kvasir-SEG and CVC-ClinicDB, respectively. This performance indicates that the proposed framework can effectively capture the main semantic and structural characteristics of polyp regions, leading to accurate segmentation on these two benchmark datasets.

In addition to the in-domain results, the generalization ability of the model was further examined on the unseen and more challenging CVC-ColonDB and ETIS-LaribPolypDB datasets, with detailed results reported in Table 3 and Table 4. These datasets provide a more demanding test setting and offer a clearer view of the robustness of the proposed method under cross-dataset evaluation.

**Figure 2:**
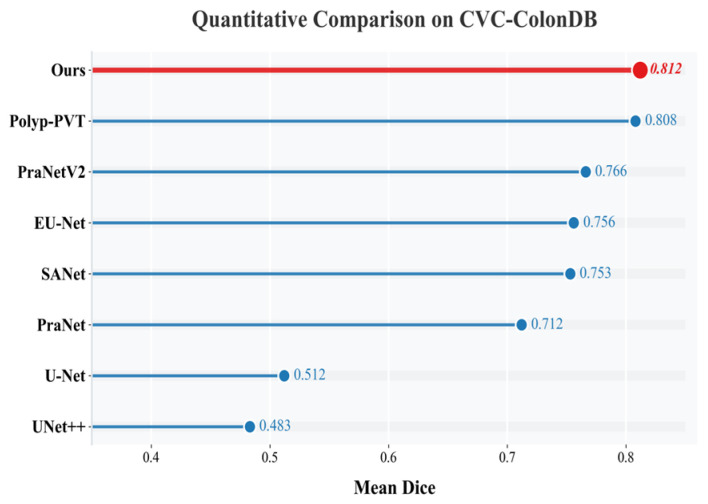
Horizontal bar comparison chart for ColonDB.

**Figure 3:**
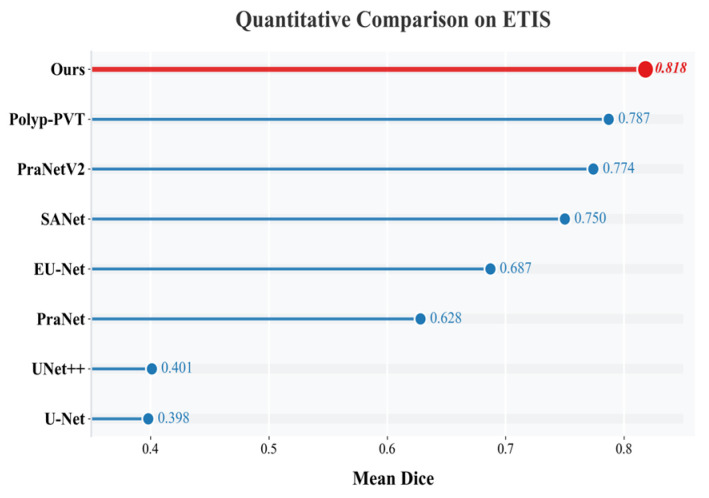
Horizontal bar comparison chart for ETIS-LaribPolypDB.

As shown in Figure 2 and Figure 3, strong performance was maintained on both CVC-ColonDB and ETIS-LaribPolypDB. On CVC-ColonDB, an mDice of 0.812 was obtained, as shown in Figure 2. On ETIS-LaribPolypDB, which contains a larger proportion of small and camouflaged polyps, the mDice reached 0.818, exceeding the values reported for Polyp-PVT (0.787) and PraNetV2 (0.774), as shown in Figure 3. Because segmentation accuracy alone is insufficient for practical deployment, computational efficiency is further analyzed in the following subsection.

### 4.5. Computational Efficiency Analysis

**Figure 4:**
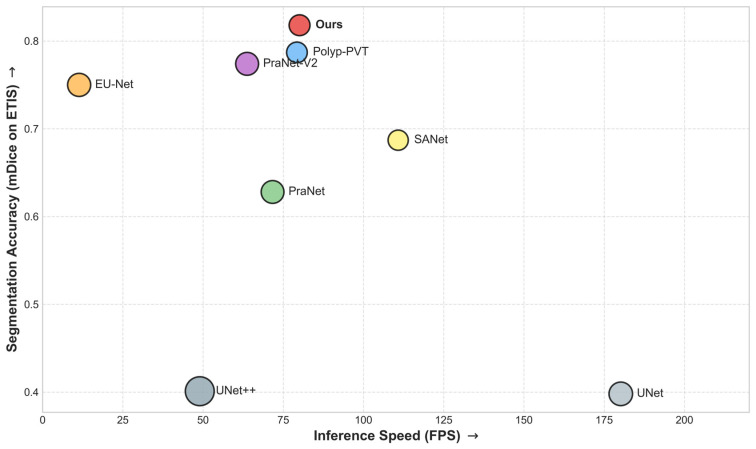
Trade-off between segmentation accuracy (mDice) and inference speed (FPS) on the ETIS dataset.

To evaluate practical applicability, Table 5 and Figure 4 present the efficiency–accuracy trade-off of DSF-BRNet and several representative baselines. In clinical endoscopy, real-time processing generally requires inference speeds above 30 FPS. Although heavily parameterized architectures such as UNet++ and EU-Net incur higher computational costs, DSF-BRNet achieves 80.08 FPS with 25.55 M parameters. Compared with PraNet-V2 and Polyp-PVT, similar or lower model complexity was maintained while competitive or better segmentation accuracy was obtained. As shown in Figure 4, the lightweight 1D convolution in the DSF module and the fixed-weight Sobel operator in the HBR module added little latency, resulting in a favorable balance between efficiency and accuracy.

### 4.6. Qualitative Analysis

**Figure 5:**
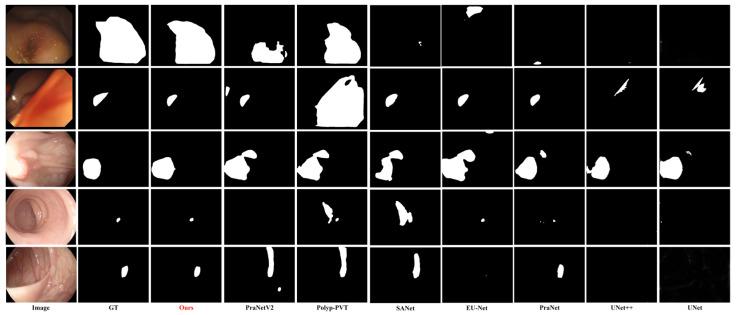
Comparison of experimental results.

To provide an intuitive comparison, the visual segmentation results of different models under challenging colonoscopy scenarios are presented in Figure 5. The selected cases encompass massive polyps with irregular shapes in the first and second rows, small polyps in the fourth and fifth rows, and flat polyps sharing similar textures with the surrounding mucosa in the third row.

As observed in the visual comparisons in Figure 5, early CNN-based models, including U-Net and UNet++, occasionally fail to completely locate the lesions, resulting in under-segmentation. Advanced models like PraNet and Polyp-PVT exhibit improved localization but can still be affected by complex background interference; for example, in the second row, Polyp-PVT and SANet segment areas of normal intestinal folds as foreground targets, resulting in false positives. In comparison, the proposed DSF-BRNet generates segmentation masks that more closely align with the ground truth. Guided by the HBR module, the contours generated by DSF-BRNet remain continuous and clear, mitigating the boundary ambiguity observed in other methods.

### 4.7. Ablation Experiments

To validate the effectiveness of the core components in the proposed DSF-BRNet, an ablation study was conducted on the Kvasir-SEG and ETIS-LaribPolypDB datasets, with quantitative results summarized in Table 6.

The baseline model achieved an mDice of 0.759 on the challenging ETIS-LaribPolypDB dataset. Adding only the HBR module yielded a marginal improvement to 0.762, suggesting that boundary refinement alone is less effective without reliable semantic alignment, since high-frequency cues may also respond to surrounding mucosal structures. In contrast, incorporating only the DSF module improved the mDice to 0.781, indicating that cross-level semantic alignment plays a more critical role in establishing discriminative feature representations. When DSF and HBR were jointly employed, the model achieved the best performance with an mDice of 0.818. These results support the effectiveness of the proposed align-then-refine framework, where DSF provides a reliable semantic basis for subsequent boundary refinement.

To further investigate the internal design of the proposed DSF module, three representative variants were additionally evaluated on the ETIS-LaribPolypDB dataset: channel-only gating, spatial-only gating, and the full dual-gated DSF.

As shown in Table 7, both the channel-only and spatial-only variants improved performance, which indicates that channel recalibration and spatial filtering are both helpful for cross-level feature fusion. The full DSF achieved the best result, showing that these two gating operations are complementary rather than redundant.

### 4.8. Visualization Analysis of the DSF Module

To provide more intuitive evidence for the role of the proposed DSF module, feature representations before and after DSF were visualized on several representative samples.

**Figure 6:**
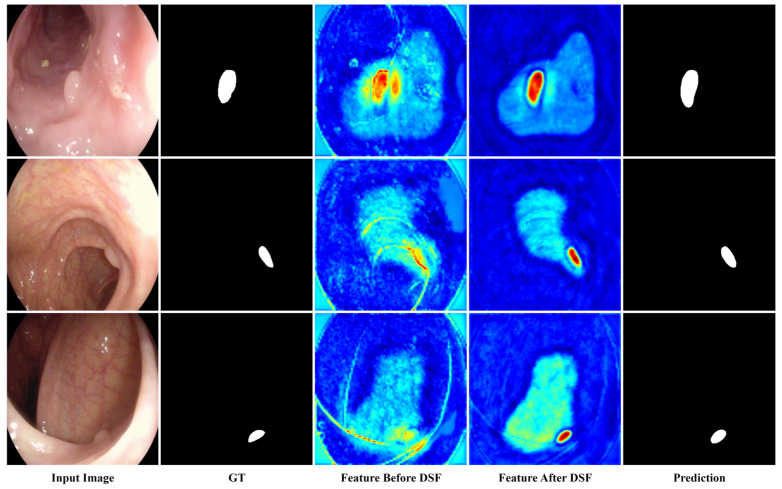
Visualization of feature representations before and after DSF.

As shown in Figure 6, before DSF refinement, the fused features still contained noticeable background responses and structural interference from surrounding mucosal patterns. After DSF processing, the activations became more concentrated on the lesion regions, while irrelevant responses were reduced. These observations suggest that DSF helps improve cross-level semantic alignment and enhances the discriminability of fused feature representations.

### 4.9. Failure Cases and Limitations

**Figure 7:**
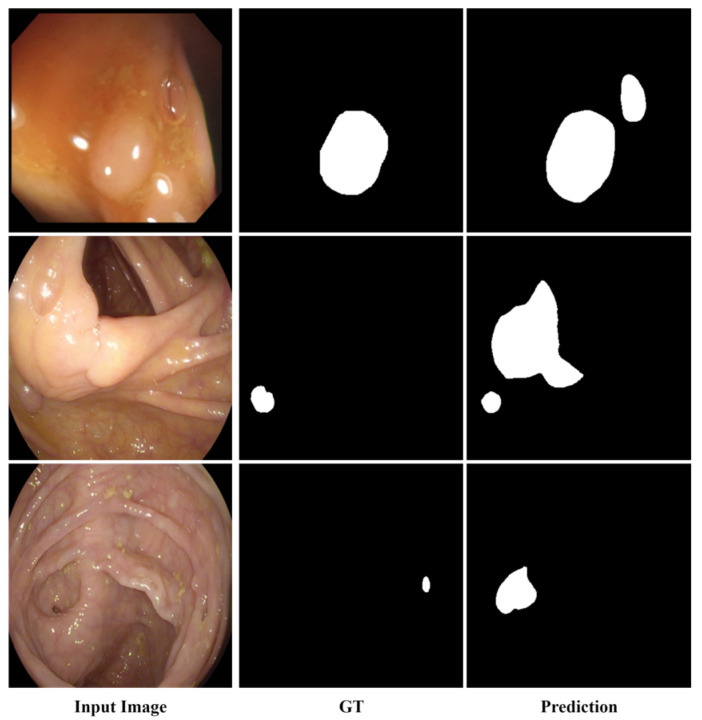
Representative failure cases of the proposed method.

Although favorable overall performance is achieved, several challenging cases remain difficult to handle. As shown in Figure 7, these failure cases are often associated with specular highlights, bubbles or debris, and visual adhesion to surrounding mucosal structures. Under such conditions, false-positive responses, boundary deviation, or over-segmentation may still occur. These observations indicate that highly ambiguous local appearance and strong structural interference remain challenging for the proposed framework.

## 5. Conclusions

A novel deep learning framework, termed DSF-BRNet, is presented for endoscopic polyp segmentation. To address complex intestinal backgrounds and boundary ambiguity, an Align-then-Refine mechanism is introduced so that semantic alignment and boundary refinement can be handled in a progressive manner.

Under this strategy, robust semantic localization is performed before precise structural delineation. The Dual-Gated Semantic Fusion module reduces information loss by avoiding dimensionality reduction and by progressively correcting spatial misalignment between cross-level features, thereby establishing a cleaner semantic foundation. The High-Frequency Boundary Refinement module then uses explicit structural gradients to refine polyp contours without relying on implicit foreground erasure.

Extensive experiments on four public benchmark datasets, including Kvasir-SEG, CVC-ClinicDB, CVC-ColonDB, and ETIS-LaribPolypDB, demonstrate competitive accuracy with favorable computational efficiency. With a real-time inference speed of 80.08 FPS and 25.55 M parameters, a favorable balance between segmentation accuracy and computational cost is achieved. These findings indicate that the Align-then-Refine paradigm may be useful for efficient polyp segmentation.

Given the favorable inference speed of DSF-BRNet, extension of the framework to video polyp segmentation will be investigated in future work. Evaluation on more diverse clinical data and further exploration of practical applicability in real-world endoscopic settings will also be pursued.

## Figures and Tables

**Table 1:** Comparative experimental results of the models on Kvasir-SEG.

Model	mDice	mIoU	$F_{\beta }^{w}$	$S_{\alpha }$	$E_{\phi }$
U-Net [8]	0.818	0.746	0.794	0.858	0.881
UNet++ [9]	0.821	0.743	0.808	0.862	0.886
PraNet [10]	0.898	0.840	0.885	0.915	0.944
EU-Net [38]	0.908	0.854	0.893	0.917	0.951
SANet [19]	0.904	0.847	0.892	0.915	0.949
Polyp-PVT [11]	0.917	0.864	0.911	0.925	0.956
PraNet-V2 [39]	0.914	0.861	0.917	0.925	0.966
**DSF-BRNet (Ours)**	**0.933**	**0.885**	**0.938**	**0.935**	**0.983**

**Table 2:** Comparative experimental results of the models on CVC-ClinicDB.

Model	mDice	mIoU	$F_{\beta }^{w}$	$S_{\alpha }$	$E_{\phi }$
U-Net [8]	0.823	0.755	0.811	0.889	0.913
UNet++ [9]	0.794	0.729	0.785	0.873	0.891
PraNet [10]	0.899	0.849	0.896	0.936	0.963
EU-Net [38]	0.902	0.846	0.891	0.936	0.959
SANet [19]	0.916	0.859	0.909	0.939	0.971
Polyp-PVT [11]	0.937	0.889	0.936	0.949	0.985
PraNet-V2 [39]	0.931	0.881	0.928	0.945	0.982
**DSF-BRNet (Ours)**	**0.943**	**0.896**	**0.939**	**0.950**	**0.993**

**Table 3:** Comparative experimental results of the models on CVC-ColonDB.

Model	mDice	mIoU	$F_{\beta }^{w}$	$S_{\alpha }$	$E_{\phi }$
U-Net [8]	0.512	0.444	0.498	0.712	0.696
UNet++ [9]	0.483	0.410	0.467	0.691	0.681
PraNet [10]	0.712	0.641	0.699	0.821	0.847
EU-Net [38]	0.756	0.681	0.732	0.831	0.863
SANet [19]	0.753	0.671	0.726	0.837	0.869
Polyp-PVT [11]	0.808	0.727	0.795	**0.865**	0.913
PraNet-V2 [39]	0.766	0.684	0.779	0.841	0.964
**DSF-BRNet (Ours)**	**0.812**	**0.728**	**0.819**	0.862	**0.967**

**Table 4:** Comparative experimental results of the models on ETIS-LaribPolypDB.

Model	mDice	mIoU	$F_{\beta }^{w}$	$S_{\alpha }$	$E_{\phi }$
U-Net [8]	0.398	0.335	0.366	0.684	0.643
UNet++ [9]	0.401	0.344	0.391	0.683	0.629
PraNet [10]	0.628	0.567	0.601	0.794	0.808
EU-Net [38]	0.687	0.609	0.636	0.793	0.807
SANet [19]	0.750	0.654	0.685	0.849	0.881
Polyp-PVT [11]	0.787	0.706	0.751	0.871	0.906
PraNet-V2 [39]	0.774	0.697	0.757	0.865	0.903
**DSF-BRNet (Ours)**	**0.818**	**0.744**	**0.803**	**0.886**	**0.988**

**Table 5:** Computational efficiency comparison of different models.

Metric	UNet	UNet++	PraNet	SANet	EU-Net	Polyp-PVT	PraNet-V2	DSF-BRNet
Params (M)	32.52	48.99	30.5	23.9	31.36	25.11	30.8	25.55
FLOPs (G)	20.24	108.83	13.15	11.34	23.15	10.02	11.23	11.37
FPS	180.17	48.99	71.67	110.81	11.40	79.26	63.74	80.08

**Table 6:** Ablation results on the Kvasir-SEG and ETIS-LaribPolypDB datasets.

Method	Baseline	HBR	DSF	Kvasir-SEG		ETIS-LaribPolypDB	
				mDice	mIoU	mDice	mIoU
1	√			0.910	0.856	0.759	0.668
2	√	√		0.916	0.865	0.762	0.687
3	√		√	0.921	0.871	0.781	0.702
4	√	√	√	**0.933**	**0.885**	**0.818**	**0.744**

**Table 7:** Additional ablation analysis of the DSF gating design on ETIS-LaribPolypDB.

Variant	ETIS-LaribPolypDB	
	mDice	mIoU
DSF-Channel	0.776	0.695
DSF-Spatial	0.775	0.690
DSF-Full	0.781	0.702

## Data Availability

The original contributions presented in this study are included in the article. The code used in this study will be made publicly available upon acceptance. Further inquiries can be directed to the corresponding author.

## Abbreviations

The following abbreviations are used in this manuscript:

DSF-BRNet	Dual-Gated Semantic Fusion and Boundary Refinement Network
DSF	Dual-Gated Semantic Fusion
GT	Ground Truth
HBR	High-Frequency Boundary Refinement
mDice	Mean Dice Similarity Coefficient
mIoU	Mean Intersection over Union
